# A Review of Novel Antioxidant Ergothioneine: Biosynthesis Pathways, Production, Function and Food Applications

**DOI:** 10.3390/foods14091588

**Published:** 2025-04-30

**Authors:** Haijing Zhang, Zheng Liu, Zhong Wang, Ziteng Lei, Yan Jia, Wei Chen, Ruoyu Shi, Chengtao Wang

**Affiliations:** 1 Beijing Advanced Innovation Center for Food Nutrition and Human Health, Beijing Engineering and Technology Research Center of Food Additives, School of Food and Health, Beijing Technology and Business University, Beijing 100048, China; haijingvv@yeah.net (H.Z.); 2330201008@st.btbu.edu.cn (Z.L.); 2431031063@st.btbu.edu.cn (Z.W.); 2431032108@st.btbu.edu.cn (Z.L.); wangchengtao@th.btbu.edu.cn (C.W.); 2 Beijing Key Laboratory of Plant Resources Research and Development, College of Light Industry Science and Engineering, Beijing Technology and Business University, Beijing 100048, China; 3 Yunnan Plateau Characteristic Agricultural Industry Research Institute, Yunnan Agricultural University, Kunming 650201, China; shiruoy@126.com

**Keywords:** ergothioneine, biosynthesis, food industry, fermentation, therapeutic applications, functional foods

## Abstract

Ergothioneine (EGT), a natural thiol compound with potent antioxidant properties, exhibits diverse biological functions, including anti-inflammatory, neuroprotective, and cardioprotective effects. Despite its promising health and food applications, current production methods, such as mushroom-based liquid fermentation, are hindered by low yields and complex processes. Advances in biosynthetic fermentation, including heterologous expression of key pathway genes and optimization of cultivation conditions, offer promising solutions to these challenges. Recent discoveries, such as the catalytic efficiency of mononuclear non-heme iron enzymes like Egt1 and EgtB, have streamlined EGT biosynthetic pathways, reducing steps and increasing yield. The compound’s active transport via the OCTN1 protein facilitates its distribution across tissues, enhancing its therapeutic efficacy and potential in functional foods. Currently employed as an antioxidant and antimelanogenic agent in aquatic products, EGT holds vast potential for broader applications in food systems. This review explores the advancements in EGT production and biosynthesis while emphasizing its prospects as a safe, versatile, and effective natural ingredient for health and industrial applications.

## 1. Introduction

Ergothioneine (EGT) is a sulfur-containing histidine derivative. Chemically, a sulfur atom is attached to the imidazole ring of histidine. It exists in solution as a tautomeric mixture of thiol and thione forms, with the thione form being more stable [1]. As a potent natural antioxidant, the lower redox potential and isomerization make EGT markedly different from glutathione (GSH) [2]. EGT was first discovered and purified by Tanret from the ergot fungus *Claviceps purpurea* in 1909 and was named trimethylbe-taine of 2-thiol-1-histidine [3]. It was subsequently shown that only certain bacteria and fungi could be able to biosynthesize EGT, such as *Neurospora crassa* [4], *Mycobacterium smegmatis* [5], *Chlorobium limicola* [6], and *Methylobacterium* strains [2]. Mushrooms have been proven to be the highest dietary source of EGT [7,8]. As research on EGT expands, its biosynthetic pathways have been progressively elucidated, revealing both aerobic and anaerobic routes to its synthesis.

The characterization of the EGT biosynthesis pathway in bacteria and fungi has made genetic engineering for the production of EGT an efficient and cost-effective manufacturing strategy. Production of ergothioneine in microbial cell factories will provide a sustainable and low-cost alternative to current manufacturing processes. The biosynthesis method for EGT preparation demonstrates markedly higher efficiency and yield compared to existing techniques for the extraction and concentration of usable fungi. More and more research attempts have been made to construct aerobic biosynthesis pathways for EGT in different hosts [9,10,11,12,13]; in addition, these different pathways highlight the complexity of EGT biosynthesis and underscore the roles of specific enzymes and their respective substrates. Scientists hope to find more efficient and rapid synthesis pathways through a homologous search of the known biosynthesis routes of EGT so that more theoretical support will be provided for subsequent research.

EGT has also been found to exist and function in the human body, but the human body cannot synthesize it and can only be incorporated into the diet. EGT ingested through the food chain does not autonomously cross the hydrophilic amphiphilic plasma membrane and requires the transport of the specific transporter EGT (also known as the transporter protein OCTN1), which is also the only biomarker of EGT activity to date [14]. Subcellular localization and real-time RT-PCR monitoring showed that EGT is distributed and stored in numerous body organs [15]. Notably, the distribution of EGT in higher animals is organ- and species-differentiated, and this variability provides key clues to the function of EGT.

EGT can scavenge reactive oxygen species (ROS) and chelate divalent metal cations such as iron and copper. Because of its potent antioxidant properties, EGT is widely used in the food industry, primarily as an antioxidant [16,17]. EGT also has a variety of biological activities, such as anti-inflammatory [18], neuroprotective effects [19], protection of the heart [20], and anti-cancer damage [21]. Even the recent epidemic of coronavirus infectious diseases (COVID-19) has an actual anti-loss effect and potential therapeutic effect [14]. Thus, increasing EGT intake through appropriate supplementation or improved diets could reduce most of the consequent risk of chronic disease and premature aging, consistent with Ames et al.’s definition of the “vitamin of life” [22]. It is also incorporated into functional foods, acting as an active ingredient with potential health benefits. Given its versatility and safety, EGT is gaining recognition for its antioxidant properties and ability to target specific tissues and exert broader biological effects.

In this review, the latest research progress on the production methods and food applications of EGT is summarized, and multiple physiological functions of EGT are discussed. In particular, we describe EGT’s antioxidant mechanism and antioxidant assays both in vivo and vitro Finally, further research and potential applications of EGT in the food industry are explored. Because the current application of EGT is mainly focused on its antioxidant effect, and its application in the food industry is more limited (primarily aquatic products research), we offer a reference for the application of EGT in the food industry.

## 2. Biosynthetic Pathway of EGT

**Figure 1:**
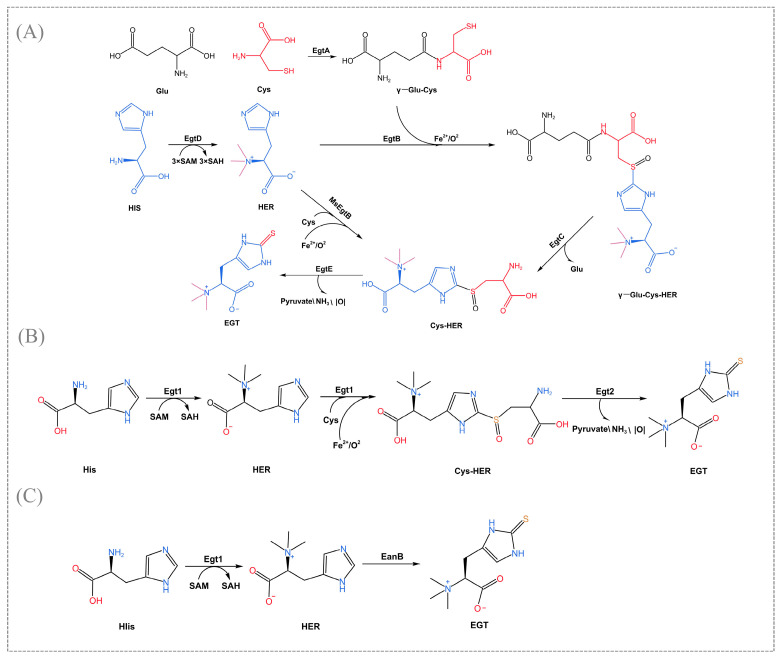
Biosynthetic pathway of ergothioneine in mycobacteria. (**A**) The bacterial pathway of aerobic synthesis pathway. (**B**) The fungal pathway of the aerobic synthesis pathway. (**C**) The anaerobic synthesis pathway.

EGT is derived from various sources but is only synthesized in fungi and bacteria. In the EGT biosynthetic pathway, especially for the heterologous production of EGT, the key step is substituting the hercynine’s ε-carbon sp^2^ C-H bond with a C-S bond [23]. Currently, known biosynthetic pathways can be classified as follows, depending on the enzyme that catalyzes the key reaction, as shown in Figure 1.

In 2010, Seeback et al. first illuminated the complex synthetic pathway of EGT in *Mycobacterium smegmatis*, which is catalyzed by the sulfoxide synthase EgtB (mononuclear non-heme iron enzyme) for the formation of an O_2_-dependent C-S bond [5]. The biosynthesis of the EgtB-pathway begins with the methylation of histidine to trimethyl histidine (TMH), followed by the EgtA (γ-glutamyl cysteine synthase) formed the synthesis of γ-glutamyl cysteine (γ-Glu-Cys) from ligating glutamate and cysteine. The next step, catalyzed by EgtB under Fe^2+^ and O_2_ conditions, was to combine TMH with γ-Glu-Cys. Some multiple intermediates are generated catalytically by EgtC (amidotransferase) and EGT (PLP-mediated C-S lyase) for the final release of EGT (Figure 1A). The homologs of egtD and egtB also exist in various prokaryotic organisms such as *Cyanobacteria*, *Actinobacteria*, the *Phylum firmicutes*, and many *proteobacteria*. Fungi have a more efficient EGT biosynthesis pathway compared to bacteria. Bello et al. identified a specific EGT biosynthetic enzyme-Egt1 for the first time in *N. crassa* [4]. The non-heme iron enzyme Egt1 catalyzes a similar but more concise EGT biosynthesis pathway that prefers cysteine as a substrate compared to glutamyl cysteine, and this change in substrate preference eliminates competition between EGT and glutathione biosynthesis. Egt1 transfers the three methyl groups from s-adenosylmethionine (SAM) to histidine to form histidine trimethylene salt. Further, it catalyzes the formation of hecynylcysteine sulfoxide from histidine trimethylene salt under Fe^2+^-O_2_ addition using cysteine as a substrate. Finally, EGT was generated by Egt2 (hercynylcysteine S-oxide lyase) catalysis (Figure 1B). Gallagher et al. first identified MaeEgtB in the pathogenic fungus *Aspergillus fumigatus*, which also uses cysteine as a substrate but is considered a different type of enzyme from those mentioned above [24]. In addition, a recent study found that EGT biosynthesis in *Methylobacterium* strains requires only three steps: EgtD→MsEgtB→EgtE [25]. MsEgtB can also use cysteine as a sulfur donor to convert histidine trimethylene salt into hecynylcysteine sulfoxide, thus reducing the EGT biosynthetic pathway from five to three steps (Figure 1A). Therefore, this particular EGT biosynthetic pathway is a more suitable platform than *Mycobacterium smegmatis* for the production of EGT by metabolic engineering.

These enzymes (EgtB, Egt1, MaeEgtB, MsEgtB), which catalyze key steps with different substrate preferences and regioselectivities, belong to varying isoforms of sulfoxide synthase. Based on the conserved amino acid sequence and catalytic residues, sulfoxide synthases can be divided into five different clades (Table 1). Crystallographic structures analysis of these enzymes revealed that the crystal structure of EgtB (type I) coordinates with the substrate γ-Glu-Cys in an octahedral iron binding site and O_2_-dependent C-S bond formation via an iron (III)-complexed thiyl radical attacking the imidazole ring of N -α-trimethyl histidine [26]. The crystal structure of MsEgtB is not clear. However, studies have shown that it has a contiguous residue Tyr with about 50% identity to CthEgtB (type II) and may have a similar structure and catalytic mechanism, such as a unique tetrameric structure and completely different residues in the binding and activation of the O_2_ activation site [27]. The type II clade employs HER and L-Cys as substrates to facilitate the direct formation of Cys-HER [28]. In addition, the enzyme Egt1 from *Neurospora crassa* belongs to the type III clade, and sequence comparison revealed that Egt1 (type III) lacks the RXXR motif but also recognizes cysteine [29]. Generally, it can be seen that sulfoxide synthase is specifically present, both in terms of chemical structure and reaction mechanism, and that each isoform must bind substrate in a markedly different manner. To unearth additional biosynthetic pathways, the structure and function of this class of enzymes need to be more fully explored. The mechanisms leading to the emergence of enzymes differing in substrate and product specificity, substrate binding modes, active site structure, and quaternary structure are still not fully and clearly explained, e.g., the enzyme from *Thermosynechococcus elongate* may constitute a type Ⅴ which neither recognizes cysteine nor accepts γ-GC as a substrate [27]. This type needs to be more characterized. Therefore, the related enzymes’ crystal structure and sequence diversity are still areas of interest.

Scientists have recently revealed the anaerobic biosynthetic pathway of EGT in the bacterium *Chlorobium limicola* [6]. In this pathway, a rhodanese domain-containing protein EanB transfers sulfur to the imidazole ring of the hercynine, and hercynine is generated by the EanA (methyltransferase), which transfers three methyl groups from SAM to histidine (Figure 1C). Experimental evidence has indicated that hercynine’s ε-carbon sp^2^ C-H bond is not activated when EanB exerts its catalytic effect but instead directly replaces the C-H bond with a C-S bond, specifically using polysulfide as a direct substrate to catalyze the trans-sulfuration reaction [30]. Tyr353 plays an important role in this trans-sulfuration reaction. Further, this enzyme mechanism is proposed based on protein crystal structure and kinetic studies. Cys412 persulphide is the key reaction intermediate, and the side chain of imidazole is activated by protonation to form the suitable intermediate IM-1. The nucleophilic attack of the persulphide leads to the formation of the C-S bond via a sequential or synergistic pathway [31]. It is also suggested that this oxidative sulfidation may occur in a wide range of bacteria. Another explanation supports the involvement of carbene (a highly reactive intermediate) in the catalysis of EanB [32]. Currently, no specific and exact mechanism has still described, and additional research could be focused on this area in the future.

The proposed synthetic pathways and mechanisms mentioned above also provide ideas for finding more diverse EGT biosynthetic pathways, and other similar biosynthetic pathways are yet to be unveiled.

**Table 1:** Five clades of sulfoxide synthases.

Types	Microbial Species	Enzyme	References
Type Ⅰ	*Mycobacterium smegmatis*	MsmEgtB	[5]
	*Mycobacterium thermoresistibile*	MthEgtB	[33]
Type Ⅱ	*C. thermophilum*	CthEgtB	[28]
	*Methylobacterium*	MsEgtB	[27]
Type Ⅲ	*Neurospora crassa*	NcrEgt1	[29]
Type Ⅳ	*Microcystis aeruginosa*	MaeEgtB	[24]
Type Ⅴ	*Thermosynechococcus elongate*	TelEgtB	[27]

## 3. Production of EGT

**Figure 2:**
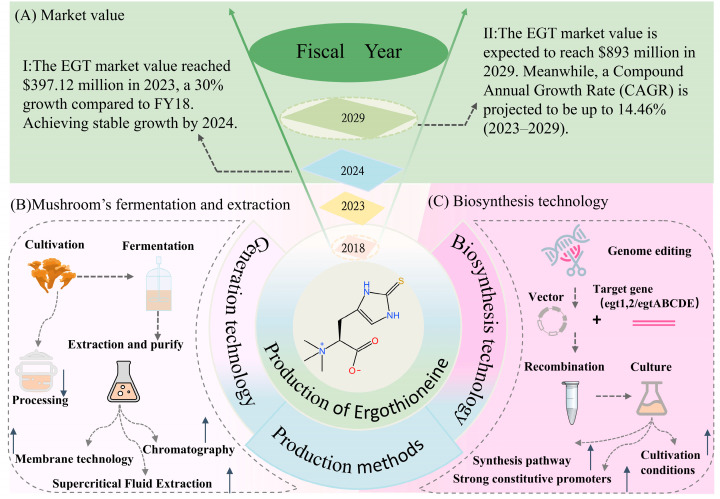
Market value and production of ergothioneine. (**A**) Schematic of the market value of ergothioneine in FY18–24 and the estimated market value in FY2029; FY: Fiscal Year. (**B**) Overview of the mushroom’s fermentation and extraction steps. Membrane technology, supercritical fluid extraction with CO_2_, and chromatography extraction techniques can increase extraction yields, and direct processing of mushrooms, such as boiling, steaming, and microwaving, is less efficient. (**C**) Biosynthesis technology synthesis pathways, strong constitutive promoters, and cultivation conditions are among the strategies to increase ergothioneine production in the microbiological incubation stage.

Ergothioneine presents considerable market potential. According to MarketWatch, the market value was approximately $397.12 million in 2023, marking a 30% increase compared to fiscal year 2018. By 2029, this market value is expected to reach $893 million, with a projected Compound Annual Growth Rate (CAGR) of up to 14.46% from 2023 to 2029. Currently, the primary market for EGT is the United States, followed by China and Europe, with the Chinese market changing faster in the past few years (Figure 2A). On the production side, North America and Europe are still the two leading global production regions. Nevertheless, the high price of pure EGT hinders the growth of the Ergothioneine industry.

The root cause of the high prices of EGT is that production yields and efficiencies are still relatively low. The most common production method of obtaining EGT is through dietary sources or extraction and separation techniques. Traditional production of EGT is mainly through biological extraction or chemical synthesis. Based on the complexity of the chemical synthesis process, the deep fermentation extraction and isolation techniques of microorganisms are more acceptable and popular. Table 2 presents several representative EGT production techniques, the extraction and isolation of ergothioneine in mushrooms, and the final EGT yield for each microorganism species.

Of all known dietary sources, mushrooms have the highest levels of EGT [40]. The EGT contents of mushrooms varied considerably between species, most of which could exceed 0.20 mg EGT/g dw. The *porcini* can produce the highest content, more than 7.27 mg EGT/g dw, which has important translational implications [41].

Relevant studies attempted to extract and isolate EGT from the main producer mushrooms and to increase the EGT yield by trying different extraction strategies (Figure 1B). Each processing step causes a loss of EGT, and various treatments (boiling, microwaving, steaming) resulted in a significant decrease in EGT concentration in mushrooms [37,42]. After boiling in water at 100 °C for 5 min, only 20% of the EGT was retained in the mushrooms. During storage, the outflow of EGT from the cells also reduces the edible value of the mushroom [37]. Therefore, many studies are interested in achieving higher utilization efficiency through refined extraction and separation techniques. Based on the above characteristics, EGT is sensitive to heat and highly soluble in water. In another study, Bhattacharya et al. studied supercritical fluid extraction with CO_2_ to extract EGT from flat mushrooms and optimized the best process variables for supercritical fluid extraction [38]. At a pressure of 21 MPa, 48 °C temperature, as the volume of co-solvent increased to 133 mL, the EGT concentration in the mushroom extract rose to 1.35 mg/g dw, whereas a decline in EGT concentration was observed beyond this range. A study following this by Cremades et al. showed that they used enzyme and 5 kDa/UF membrane technology to obtain further EGT-enriched mushroom aqueous extracts [39]. This work effectively removed high molecular weight fractions (soluble proteins, amino acids, peptides, oligopeptides, low a-glycans, etc.) in the low cut-off range. It reached an 82.27% total recovery rate of EGT by combining with the vacuum concentration technique. These extracts can be incorporated into solid or liquid food products or used as food antioxidants, but the yield concentration is far below the market demand. It is reported that the concentration of EGT is only 5.50 ± 0.34% after drying and lyophilization [43].

Although some techniques, such as ion-exchange chromatography technology, can further increase the yield, this step significantly increases the cost of production. It is more suitable for nutraceuticals and pharmaceuticals requiring high concentrations of EGT [44]. Besides process issues, mushroom cultivation faces problems such as low component yields and spontaneous denaturation associated with substrates, which also limit the large-scale production of EGT. So, there is an urgent need to develop a more efficient production method.

Fortunately, the EGT biosynthetic pathway and related enzymes have become more apparent in recent years. Chen et al. integrated the CmE1B, CmEgt2, and EgtD, of *C. militaris* into *E.coil* by combining high-intensity promoters, then they modified the genes in *C. militaris* by introducing the antioxidant enzyme Gpx, resulting in a maximum EGT content of 2.5 g/kg [10]. Although the *C. militaris* overexpression strain did not resolve substrate degeneration, expression of the EGT synthase gene restored the degenerating strain’s ability to produce many components, suggesting that metabolic regulation of EGT may release symptoms of mushroom degeneration. Indeed, EGT proved promising in ameliorating this inherent problem of mushroom degeneration [45].

In addition to introducing a highly expressed promoter, Wang et al. enhanced the supply of the three precursors amino acids of EGT, histidine, methionine, and cysteine. Eventually, the optimized strain E1-A1-thrA-serA successfully produced 548.75 mg/L and 710.53 mg/L EGT in glucose inorganic salt medium and rich medium, confirming that enhanced precursor supply is an effective control strategy [11]. By heterologously overexpressing the egt1 and egt2 genes of Neurospora crassa, *A. oryzae*’s yield is up to 231 mg/kg, which is comparable to mushroom content (16 to 417 mg/kg wet weight), 20 times higher than the wild type strain [10]. Likewise, yeast species are commonly used hosts in food production, such as oleaginous *yeast*, and *Saccharomyces cerevisiae*, which can produce EGT from 150 mg/L to 600 mg/L [12,46]. *Escherichia coli* (*E. coli*) is also commonly used as a chassis cell. In *E. coli*, 24 mg/L, secreted EGT was produced by expressing the egtBCDE gene from *Mycobacterium pubescent* and optimizing the medium composition [34]. In the latest study, Liu et al. recombinantly constructed the EGT biosynthesis pathway in *E.coli* from *Trichoderma reesei*, which allowed co-expression of the genes (tregt1 and tregt2) to produce 70.59 mg/L of EGT at the level of the vibrating flasks. The extracellular EGT yield reached 4.34 g/L by batch replenishment fermentation after incubation for 143 h in a 2 L tank fermenter [36]. The production cycle was shortened, and the yield increased, which is the highest level of EGT production reported to date. It can be seen that the host is also particularly important for the production of EGT. Although *E. coli* is widely accepted in Europe and the United States as a chassis cell application in the food industry, its safety and disadvantages should be fully considered.

In particular, plant surface-symbiotic *Methylobacterium* species can accumulate large amounts of EGT, which almost all *Methylobacterium* type strains can synthesize. Alamgir et al. successfully increased the yield of EGT by optimizing the culture conditions of high-yielding strains (*M. aquaticum* strain 22A) [6]. Since the high cell density of *Methylbacterium*, the production level overwhelms that of the most productive mushroom when given sufficient carbon and nitrogen sources and supplements. Compared to traditional EGT synthetic materials, we are interested in further developing more efficient fermentative production using *Methylobacterium* species and methanol as a cheap feedstock.

In summary, the biosynthesis of EGT has a lower cost, shorter fermentation period, high yield, and green environment, which gives it a broad development prospect (Figure 2C). Thus, the biosynthesis of EGT is an effective method for high-yield production.

## 4. Functions and Applications of EGT

**Figure 3:**
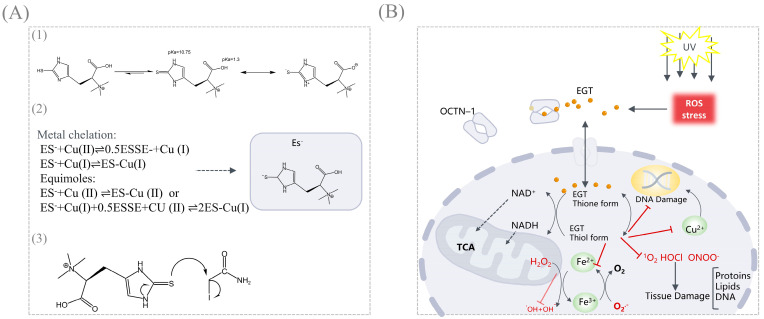
Antioxidant mechanism of EGT. (**A**) (**1**) Thiol/thione tautomerism and thiol ionization of EGT, and shows the dissociation constants (p*K*_a_) of the main proton donor functional groups. (**2**) The mechanism by which EGT and copper bind to form chelates in solution or in equimoles of EGT is different. (**3**) Nucleophilicity of the thione form of EGT is attributed to electron donation from the conjugated nitrogen atoms. (**B**) Metabolism of EGT in the cell: when the cell is stimulated by exogenous unfavorable factors such as exogenous ROS or UV-induced oxidative damage and inflammation, EGT will be rapidly transported to the damaged site by the transporter protein OCTN1 and transmembrane transported to the intracellular to occur a series of antioxidant reactions to protect the cells of the different subcellular organelles within the cell. EGT rapidly binds to Cu^2+^, reduces DNA damage known to be mainly caused by Cu^2+^, and effectively scavenges intracellularly generated ROS (H_2_O_2_, O^2−^, OH, O_2_, HOCl, ONOO^−^).

EGT has been widely used as a naturally potent oxidant for over a century, and its antioxidant effects have been well evaluated. In an aqueous solution, EGT exists as a tautomer between its thiol and thione forms (Figure 3(A1)), but at physiological pH, it exists predominantly as a thione [1]. Nucleophilicity of the thione form of EGT is attributed to electron donation from the conjugated nitrogen atoms (an example reaction with iodoacetamide is depicted, Figure 3(A3)) [47]. Structurally, it is structurally (C=S) more stable and does not autoxidize by Fenton reaction as glutathione (GSH) [48]. Another aspect is that the C=S double bond of the thione is linked to the π-conjugated structure of the imidazole ring so that the lonepairelectrons of electrons (3p orbitals) on the sulfur atom can be highly delocalized, lowering the oxidation potential [49,50]. The standard redox potential for the EGT thiol-disulfide couple is −0.06 V, whereas other naturally occurring thiols typically range from −0.2 to −0.32 V (glutathione is −0.25 V) [51]. Moreover, the redox chemistry of EGT is spontaneous and rarely dependent on enzyme availability, as the thiol interconversion isomerism permits the formation of disulfide bonds by oxidation and thus interconversion equilibrium, which may account for its strong resistance to autoxidation. Combining these two properties, EGT confers greater stability under physiological conditions, namely, antioxidant properties with a wide range of applications.

Antioxidant assays can be categorized into five mechanistic pathways: Single electron transfer (SET), hydrogen atom transfer (HAT), hydrogen atom transfer via metal chelation, ROS/RNS Scavenging activity/lipid oxidation [52,53,54]. The antioxidant properties of EGT were evaluated mainly by the following two aspects: ROS/RNS Scavenging activity and metal chelation (Taking Cu^2+^ as an example [55], in Figure 3(A2)). On the one hand, EGT acts as a potent scavenger of ROS/RNS in the human body. On the other hand, EGT can easily chelate with divalent metal ions, such as Fe^2+^, Cu^2+^, and Zn^2+^, to form inactive ergothioneine–metal complexes, which limit the metal ion reactions and thus reduce the involvement of these metal ions in the formation of ROS in vivo (Figure 3B).

Various in vivo and in vitro analytical methods have been developed to evaluate the EGT antioxidant properties (Table 3). At the cellular level, Aruoma et al. tested 5 mm EGT in a human neural hybridoma cell line (N-18-RE-105), which significantly enhanced this protective effect by inhibiting hydrogen peroxide (H_2_O_2_), and this study suggests that EGT could be used in vivo as a non-toxic thiol-buffered antioxidant and could be applied to pharmaceutical formulations that require oxidative stability [56]. Liu et al. demonstrated that EGT from culinary, medicinal mushrooms chelates metal ions and is very effective in scavenging H_2_O_2_, O^2−^, and hydroxyl radicals (OH) [18]. A more significant proportion of these experiments were in vitro. However, some studies point to the basis for the in vitro experiments being ineffective in vivo, so further confirmation of the antioxidant effects of EGT in vivo is needed in the future [57,58].

Furthermore, more and more studies have recently focused on the value of edible mushrooms in neurodegenerative diseases. Examples include Alzheimer’s disease, depression, Parkinson’s disease, and spinal cord injury. By dietary supplementation of H. erinaceus primordium (He_2_ strain) extracts containing high levels of EGT to aging-stage mice for 8 months, researchers significantly reduced cerebellar locomotor decline and oxidative stress in the experimental mice. Therefore, they demonstrated that EGT in medicinal mushrooms exerted neuroprotective and preventive effects to ameliorate, attenuate, or recover from age-dependent injuries [63]. Further, by intravenously injecting synthetic EGT PET radioligand into a mouse model of Alzheimer’s disease, the researchers observed that EGT crosses the brain barrier and distributes in several regions of the mouse brain, with the signal remaining firm 30 min after injection and at a higher level than that of normal mice, further demonstrating the therapeutic effect of EGT [64]. In addition, external uptake of EGT can accumulate rapidly in the heart through the bloodstream, which could mean it may be protective of the cardiovascular system [20]. Cheah et al. noted the potential of EGT supplementation to prevent cardiac dysfunction in a mouse model of doxorubicin-induced cardiotoxicity and found it to be significantly toxicoprotective [19]. Not only did EGT not interfere with the chemotherapeutic effects of doxorubicin, but tumors were no longer exacerbated in the administered mouse model [21]. The cardioprotective effects of EGT may result from a combination of other functions (antioxidant, anti-inflammatory, and cardiovascular protection). EGT is indeed prominent in the anti-inflammatory profile.

In particular, EGT has been shown to have some therapeutic effect on the recent epidemic of coronavirus infectious disease (COVID-19), which not only reduces pulmonary fibrosis but also significantly reduces the damage caused by COVID-19 in several organs, including the kidneys, liver, gastrointestinal tract, and testes [65]. This therapeutic effect is attributed to the active absorption and accumulation of EGT in high concentration in the body, which is transported through the transmembrane protein OCTN1 and distributed in most tissues and organs of the body (liver, kidneys, spleen, lungs, testes, eyes, red blood cells, and the brain), and the feedback mechanism by which EGT regulates the expression of OCTN1, which further leads EGT to the site of tissue and organ damage, allowing it to exert its powerful beneficial functions [14]. It allows EGT to perform robust, beneficial functions. These studies provide strong evidence for the practical application of EGT. Taken together, EGT has a powerful function that revolves around antioxidant function, and a series of other biological functions such as anti-inflammation, prevention, and treatment of neurodegenerative diseases, cardiovascular protection, cancer protection, and treatment of diabetes, etc., can be exerted in the human body through exogenous dietary intake. Thus, it is also incorporated into functional foods, acting as an active ingredient with potential health benefits.

In the food industry, EGT also has a broad application prospect. Currently, the phenomenon of blackening of aquatic products caused by phenol–alcohol polymerization is one of the urgent problems in the food industry. Black spots usually occur in the cephalothorax of crustaceans during post-harvest storage and spread to other parts of the animal; they also occur in damaged or senescent parts of mushrooms and plants [66]. Black spots are not harmful to the product, but they also affect customer acceptance and cause significant losses to the commercial quality and value of the food product. To address this problem, Encarnacion et al. immersed live adult shrimp in a 0.5% *w*/*v* mushroom extract solution, significantly reducing oxidase activity in shrimp hemolymph [17]. Reduced expression of the propoxyphenol oxidase (proPO) gene in hemocytes was demonstrated by vitro experiments, suggesting that the extract blocked the activation of the proPO cascade, and this study proved that EGT plays a significant role in mushroom extracts and can be used as a natural alternative to antimelanogenic and antioxidant agents in food. Similarly, dietary supplementation of shrimp with aqueous mushroom extract by feed showed lower expression of transcripts of the proPO gene in shrimp hemocytes in EGT-treated HLS [16]. Lipid oxidation in the flesh of Kuruma shrimp was also effectively controlled, and the same effect of controlling melanosis and lipid oxidation in shrimp muscle could be achieved. These biochemical interventions could have the same effect on other types of aquatic products [67,68]. Although the use of EGT as an antimelanogenic agent is mainly limited to seafood products, with the in-depth study of the functional mechanism of EGT, research on other types of food products has been carried out. Ye et al. used extracts of *F. velutipes* in emulsified sausages, and the EGT in the extracts showed good scavenging activity against the free radicals DPPH (90.2%), ABTS (96%), and hydroxyl groups (87.6%) [69]. It was proved that the addition of EGT-rich fuzzy chicory extract to sausages could effectively prevent lipid and protein oxidation during refrigeration, and the flavor sensation could be enhanced, so EGT is a strong candidate as a natural antioxidant for meat products. Finally, the ultrafiltration products of EGT extracted from the edible mushroom *Agaricus bisporus* are effective scavengers of DPPH free radicals and inhibitors of linoleic acid oxidation, suggesting that they can be used as natural antioxidants in lipid-based foods [70]. Based on the powerful physiological functions of EGT, edible fungi or fungal extracts enriched with this component are expected to be used as functional food ingredients.

**Figure 4:**
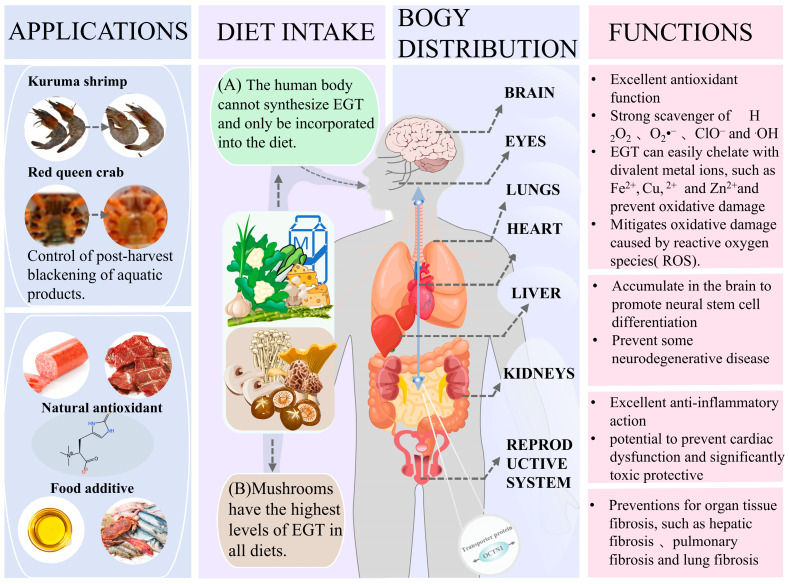
Functions and applications of ergothioneine.

The U.S. Food and Drug Administration (FDA) granted GRAS approval for EGT in California and allowed EGT as a nutritional supplement added to pastries and beverages. In 2017, the European Union (EU) proposed to evaluate the safety of synthetic EGT as a novel food additive intake (for infants and young children, pregnant women, and breastfeeding mothers) from dietary food intake. The EU proposes that synthetic EGT is safe for infants, young children (i.e., toddlers), and pregnant and breastfeeding women and that the maximum daily intake (MDI) can be referenced as infants 2.82 mg/kg body weight (bw) per day, 3.39 toddlers 1.31 mg/kg bw per day, and adults (including pregnant and breastfeeding women) 1.31 mg/kg bw per day [71]. It is important to note that EGT has beneficial effects within a specific dose range, and the finding by Petermann et al. that consumption of EGT-rich mushrooms increased the risk of Crohn’s disease (CD) in individuals carrying the OCTN1 variant single nucleotide polymorphism informs the development of the new standard [72] (Figure 4).

## 5. Conclusions and Perspectives

In conclusion, EGT is a highly efficient, safe, and stable natural antioxidant with significant potential for broad application. However, its current production levels remain limited, necessitating the development of more efficient and high-yield production methods to meet the growing market demand. Edible mushrooms, known for their high EGT content, are currently the primary source of commercial production via liquid fermentation processes. While this method has proven effective, it presents several challenges, including long cultivation periods, substrate instability, and complex extraction protocols. Biosynthetic fermentation has emerged as a promising alternative to address these limitations. Strategies such as heterologous expression of key synthesis pathway genes, enhanced precursor supply, batch replenishment fermentation, and optimized cultivation conditions have demonstrated the potential to improve production efficiency significantly.

EGT biosynthesis pathways are categorized into aerobic and anaerobic mechanisms, with notable enzymatic processes such as the C-S bond oxidation catalyzed by mononuclear non-heme iron enzymes (e.g., Egt1 and EgtB). Discovering more efficient biosynthetic pathways, such as using enzymes like MsEgtB, which reduces the bacterial synthesis pathway from five steps to three, could enhance production yields. A deeper understanding of these pathways and the mechanisms underlying key transitions in EGT biosynthesis will advance production technologies and ensure safety and efficacy for human and industrial applications.

Beyond production, the diverse biological functions of EGT—including its antioxidant, anti-inflammatory, neuroprotective, anti-carcinogenic, and cardioprotective properties—highlight its significant potential for health and wellness applications. EGT’s ability to be transported to multiple tissues and organs via the transmembrane protein OCTN1 underscores its utility as a dietary supplement and functional ingredient. Its current applications in the food industry, particularly as an antioxidant and antimelanogenic agent for aquatic products, showcase its practical benefits. Expanding the scope of EGT applications to a broader range of food products will enable more comprehensive and effective utilization of this compound. Through continued research and innovation, EGT’s full potential can be realized, benefiting both the functional food and food industries.

## Figures and Tables

**Table 2:** Production techniques and enhancement methods of Ergothioneine.

Microorganism Species	Experimental Methods	EGT Yield	Reference
*Mycobacterium pubescent*	Using Escherichia coli as an alternative host to express the egtBCDE gene from *M. pubescent* for EGT production	17 ± 1– 24 ± 4 mg/L	[34]
*Methylobacterium aquaticum*	Genetic modification of strain 22A, deletion of the histidine metabolizing enzyme gene hutH, introduction of the genome egtBD, and optimization of cultivation conditions	7 mg/g dw	[6]
*Saccharomyces cerevisiae*	Investigating the effect of amino acid supplementation of the medium and altering the nitrogen metabolism of *S. cerevisiae* with knock-out of TOR1 or YIH1	598 ± 18 mg/L	[35]
*Neurospora crassa*	Using *Aspergillus oryzae* as an alternative host for EGT production	124.5 ± 5.0– 231.0 ± 1.1 mg/kg dw	[9]
	Using Crabtree-negative, oleaginous yeast *Yarrowia lipolytica* as an alternative host for EGT production	1.63 ± 0.04 g/L	[12]
	Using *Escherichia coli* as an alternative host for EGT production, and enhancing the supply of the three precursors, amino acids of EGT	95.58 ± 3.2– 548.75 mg/L	[11]
*Trichoderma reesei*	Co-expression of EGT biosynthesis genes of *T.reesei* (tregt1 and tregt2) in *E.coli*, then fed-batch fermentation	0.89–4.34 g/L	[36]
*Agaricus blazei Murill*	Boiling, steaming, and microwaving extract treatments	0.66–2.0 g/kg dw	[37]
*Pleurotus ostreatus*	Supercritical fluid extraction with CO_2_ to extract EGT	0.90–1.35 mg/g dw	[38]
*Agaricus bisporus*	Using enzyme and 5 kDa/UF membrane technology, combined with a concentration step	0.323 ± 0.012 −118.63 ± 4.11 g/kg dw	[39]
*Cordyceps militaris*	Excavating the EGT synthetases CmE1B and CmEgt2 of C.militaris into the genome and using a strong promoter to modify the EGT synthesis pathway further.	0.511 ± 0.040– 2.485 ± 0.051 g/kg dw	[10]

**Table 3:** EGT in vivo and in vitro analytical antioxidant assays.

Mechanism (Category)	Assay	Technology	Results	Reference
	Thiobarbituic acid (TBA) method	In this assay, TBA and trichloroacetic acid are mixed with the sample solution, placed in the hot water bath for 10 min, centrifuged in the solution, and the supernatant absorbance activity is measured at 550–560 nm	EGT inhibited the peroxidation of arachidonic acid by mixed systems of myoglobin or hemoglobin and H_2_O_2_	[59]
ROS/RNS Scavenging activity/lipid oxidation	Hydroxyl radical scavenging activity (deoxyri-bose assay)	Competitive inhibition of deoxyribose degradation by EGT is used to measure its scavenging capacity, and solubilization studies with pulsed radiation are used to calculate rate constants for the reaction of OH with scavengers	Used to scavenge hydroxyl radicals (OH), proven EGT has strong scavenging power	[59]
	Peroxynitrite radical scavenging activity	The addition of ONOO- to α_1_AP at pH 7.4 causes the loss of its elastase-inhibitory capacity, EGT Competitive inhibition of ONOO^−^, and its scavenging capacity measured by enzyme activity	Used to scavenge hydroxyl radicals, peroxynitrite (ONOO^−^), proven EGT has strong scavenging power	[60]
ROS/RNS Scavenging activity /lipid oxidation	Total oxyradical scavenging capacity (TOSC)	This assay is based on the reaction of artificially generated oxyradicals with α-keto-γ-methiolbutyric acid (KMBA), which is completely oxidized to ethylene. EGT competitively inhibits ethylene production, and the antioxidant capacity of EGT was determined by measuring the amount of total ethylene	EGT was the most active ROS scavenger agent as compared to GSH, trolox, and uric acid. The scavenging capacity, anti-ROO· (5.51 ± 0.1 units), anti-hydroxyl radicals (0.34 ± 0.09 units), anti-peroxynitrite (5.2 ± 1 units), anti-peroxyl radicals (5.53 ± 1.27 units)	[54]
	Determination of single-line oxygen scavenging capacity	This assay determines the EGT scavenging capacity by measuring the time of decay of single heavy-state molecular oxygen phosphorescence at 1270 nm in D_2_O	EGT is the most active singlet oxygen quencher of the compounds studied in the present experiments, and at the normal physiological pH K_0bs_ = 2.3 × 10^7^	[60]
Metal chelation	Metal-complexes of ergothioneine (Fe^2+^, Cu^2+^, Zn^2+^)	The interaction between EGT and divalent metal ions has been investigated by means of potentiometric titration, visible and near-infrared spectra	Copper has the highest complex formation constant	[61]
In vivo	Determination of total lipid content	This assay uses the iron complex of the chelating agent nitrilotriacetic acid (Fe-NTA), based on the profile of polyunsaturated fatty acids at 264 nm for the assessment of antioxidant actions in vivo	Supplementation with EGT not only protects the organs against lipid peroxidation but also conserves the consumption of endogenous glutathione and a-tocopherol	[62]

## Data Availability

Not applicable.
